# Understanding nursing students’ acceptance of generative AI-assisted e-picture book creation in pediatric health education

**DOI:** 10.1186/s12912-026-05191-w

**Published:** 2026-08-12

**Authors:** Ching-Yi Lai, Li-Yun Tsai, Ya-Hui Tseng, Wei-Sho Ho, Wan-Yun Hsu

**Affiliations:** 1 Department of Nursing, Central Taiwan University of Science and Technology, No. 666, Buzi Rd., Beitun Dist, Taichung City, 406053 Taiwan; 2 School of Nursing, National Taipei University of Nursing and Health Sciences, No. 365, Mingde Rd., Beitou Dist, Taipei City, 112303 Taiwan; 3 Department of Electrical and Mechanical Technology, National Changhua University of Education, No.2, Shi-Da Rd, Changhua City, 500208 Taiwan; 4 Graduate Institute of Technological and Vocational Education, National Changhua University of Education, Bao-Shan Campus, No.2, Shi-Da Rd, Changhua City, 500208 Taiwan; 5 NCUE Alumni Association, National Changhua University of Education, Jin-De Campus, No. 1, Jinde Rd, Changhua City, 500207 Taiwan; 6 International Medical Service Center, Taichung Veterans General Hospital, No. 1650, Sec. 4, Taiwan Blvd., Xitun Dist, Taichung City, 407219 Taiwan

**Keywords:** Nursing students, Generative artificial intelligence, E-picture books, Pediatric health education, Technology acceptance model

## Abstract

**Background:**

Generative artificial intelligence (AI) is increasingly integrated into health professions education; however, limited research has examined nursing students’ acceptance of generative AI as a task-based creative tool for developing child-centered health education materials. In pediatric care, translating complex medical information into developmentally appropriate and emotionally supportive formats remains a critical yet underexplored competency.

**Objective:**

This study aimed to examine nursing students’ acceptance of using generative AI to create pediatric health education e-picture books and to identify associated factors based on the Technology Acceptance Model.

**Methods:**

A cross-sectional study was conducted with 80 nursing students from a private university in central Taiwan. Participants engaged in a structured learning activity using generative AI to develop pediatric e-picture books prior to completing a TAM-based questionnaire. Actual use was operationalized as participants’ self-reported engagement during the AI-assisted e-picture book creation activity. Data were collected between January and December 2025 and analyzed using descriptive statistics, Pearson correlation analysis, and multiple linear regression.

**Results:**

Nursing students demonstrated moderate to high levels of acceptance. Perceived ease of use was strongly associated with perceived usefulness (β = 0.878, *p* < 0.001). Both perceived usefulness and perceived ease of use were significantly associated with attitudes toward use, which was strongly associated with behavioral intention (β = 0.902, *p* < 0.001). Behavioral intention showed a very strong association with actual use (β = 0.971, *p* < 0.001); given the cross-sectional design, these findings reflect statistical association rather than causal pathways, and the exceptionally high behavioral intention–actual use correlation also suggests limited discriminant separation between these two constructs. Additionally, year of study was significantly associated with perceived usefulness, with first- and third-year students reporting lower scores than fourth-year students (*p* < 0.05).

**Conclusions:**

This study provides novel empirical evidence on nursing students’ acceptance of generative AI in pediatric nursing education. Findings are consistent with the applicability of TAM in this task-based learning context, pending confirmation through structural equation modeling in larger, more balanced samples, and suggest that generative AI may serve as a useful educational tool in pediatric health education.

## Introduction

Hospitalization is often a stressful experience for children due to unfamiliar environments, medical procedures, and interactions with healthcare professionals [1]. In pediatric settings, providing developmentally appropriate health education is essential to reduce anxiety and support children’s understanding of medical experiences [2, 3]. Picture books are widely used as educational and supportive tools because they combine illustrations with simple language to explain healthcare information in a child-friendly manner [4, 5]. Studies have shown that picture books can facilitate communication among healthcare providers, children, and families while reducing fear and enhancing understanding [6]. However, developing traditional pediatric health education picture books is often time-consuming and resource intensive. Tsao et al. (2017) reported that creating picture books for intravenous injection education required approximately six months [7]. In addition, healthcare professionals may encounter challenges related to illustration design, storytelling, and visual communication skills.

With the advent of the artificial intelligence (AI) era, the emergence of generative AI has gradually addressed these challenges. Generative AI, including tools such as ChatGPT, Gemini AI, and Copilot, is capable of producing text and generating novel images for creative purposes based on user prompts [8]. In the context of picture book creation, generative AI enables the rapid production of high-quality illustrations and engaging narratives tailored to diverse healthcare needs. This approach not only improves the efficiency of creating pediatric medical care e-picture books but also enhances children’s attention and understanding of complex medical information.

For Generation Z nursing students (born between 1997 and 2012), competencies related to understanding and applying AI have become increasingly important in digitally mediated learning environments. Integrating AI technologies into nursing practice may enhance professional competence and support high-quality and efficient care delivery [9]. However, concerns have also emerged regarding the use of generative AI in education, particularly related to plagiarism, academic integrity, and the reliability of AI-generated content [10]. In response, some universities have begun establishing guidelines ranging from restricted to structured use to define appropriate boundaries for AI integration in educational settings [11]. Prior research suggests that understanding users’ technology acceptance is essential for evaluating the implementation and sustainability of emerging technologies [12]. Despite the growing use of generative AI in education, limited research has examined nursing students’ acceptance of using generative AI as a creative tool for developing pediatric health education materials. Therefore, this study aimed to investigate nursing students’ acceptance of using generative AI to create pediatric health education e-picture books and identify factors influencing such acceptance.

### Literature review

Hospitalization poses multifaceted challenges for children, encompassing physical discomfort, emotional distress, and environmental unfamiliarity. Therapeutic play has been widely recognized as an effective intervention to mitigate these negative experiences. Through activities such as drawing, storytelling, and role-playing, children are able to express emotions, gain a sense of control, and better process medical experiences. Empirical studies have consistently demonstrated that play-based interventions contribute to reduced anxiety levels and improved psychological adaptation among hospitalized children [1, 3].

Among various play-based strategies, picture books have emerged as a particularly effective medium in pediatric healthcare settings. By integrating visual and narrative elements, picture books facilitate children’s understanding of complex medical procedures in a developmentally appropriate manner. Prior studies have shown that picture books can reduce fear and anxiety, improve cooperation during medical procedures, and enhance treatment adherence [4, 5]. Furthermore, picture books serve as a communication bridge between healthcare providers, children, and families, supporting more effective health education delivery [6]. However, the production of high-quality pediatric medical picture books remains resource-intensive and time-consuming. Traditional development processes require interdisciplinary collaboration, including expertise in healthcare, illustration, and storytelling. As a result, the scalability and accessibility of such educational materials are often limited in clinical practice [7].

The emergence of generative AI offers new opportunities to address these limitations. Generative AI technologies can rapidly produce text and images, enabling the creation of customized educational materials with minimal time and resource investment. In nursing education, such tools have the potential to support innovative teaching strategies, enhance student engagement, and facilitate the development of patient-centered educational resources. Nevertheless, the integration of generative AI into education also presents challenges. Concerns regarding accuracy, misinformation, ethical use, and academic integrity have been raised, highlighting the need for appropriate guidance and oversight [10, 11]. In addition, while existing studies have explored general attitudes toward AI, there remains a lack of empirical research examining how nursing students accept and utilize generative AI in specific, task-oriented learning contexts, such as the development of pediatric health education materials.

### Theoretical framework

The Technology Acceptance Model (TAM), developed by Davis (1989) [13] and grounded in the Theory of Reasoned Action [14] and the Theory of Planned Behavior [15], provides a theoretical framework for explaining users’ acceptance of new technologies [16]. TAM consists of five core constructs: perceived ease of use (PEOU), perceived usefulness (PU), attitude toward use (ATU), behavioral intention to use (BI), and actual use (AU), and delineates the relationships among these constructs [17, 18]. According to TAM, PEOU and PU are the primary determinants of technology acceptance, with PEOU influencing PU, which in turn shapes users’ ATU, BI, and AU [13]. In addition, external variables, such as demographic characteristics, may influence users’ perceptions of PEOU and PU [13, 19]. Previous studies have applied TAM to examine how users’ perceptions of technology influence attitudes and intentions, ultimately predicting AU, and the model has been widely used to assess students’ acceptance of educational technologies [20]. Factors such as age, gender, academic year, daily computer use, and frequency of generative AI use have also been examined as variables influencing technology acceptance [21].

Based on this theoretical framework, the present study employed TAM to investigate nursing students’ acceptance of using generative AI to create pediatric medical care e-picture books and to identify associated influencing factors. The findings are expected to provide empirical evidence to inform future nursing education strategies and the clinical application of generative AI–assisted health education tools.

## Methods

### Design and study participants

This cross-sectional study examined nursing students’ acceptance of using generative AI to create pediatric medical care e-picture books and identified associated influencing factors based on the TAM. Participants were recruited using a convenience sampling method from undergraduate nursing students at a private university in central Taiwan. Eligibility criteria included: (a) being a nursing student aged 18 years or older, (b) the ability to communicate in Mandarin or Taiwanese, and (c) completion of courses in Human Development and Pediatric Nursing. Students who lacked basic computer operation skills were excluded. Data were collected between January and December 2025. Sample size estimation was conducted using G*Power version 3.1.9.2 [22], with the significance level set at α = 0.05, statistical power at 0.80, and an effect size of 0.30. The analysis indicated that a minimum sample size of 64 participants was required. To account for an anticipated attrition rate of approximately 20%, a total of 80 participants were recruited. Because the regression models estimated in this study included up to eight predictors (background covariates together with TAM antecedents), the achieved sample of 80 was also evaluated against Green’s heuristic for regression sample size [23]: *N* ≥ 50 + 8 m for testing the overall model and *N* ≥ 104 + m for testing individual predictors, where m is the number of predictors.

### Instruments

#### Generative AI to create pediatric medical care e-picture books

In this study, generative AI–assisted design tools were integrated into a learning intervention to support nursing students in developing pediatric medical care e-picture books. Gemini AI *(Google)* was selected as the primary platform for its capacity to transform complex medical concepts into child-friendly visual materials, including illustrations, charts, and narrative-based content. Through AI-assisted image generation and layout design, potentially anxiety-provoking pediatric procedures were presented in an age-appropriate and visually engaging manner, facilitating comprehension and reducing cognitive burden. In addition, the platform enabled story-based and case-oriented narratives, allowing medical procedures to be embedded within relatable contexts that are particularly effective for pediatric health education.

**Fig. 1:**
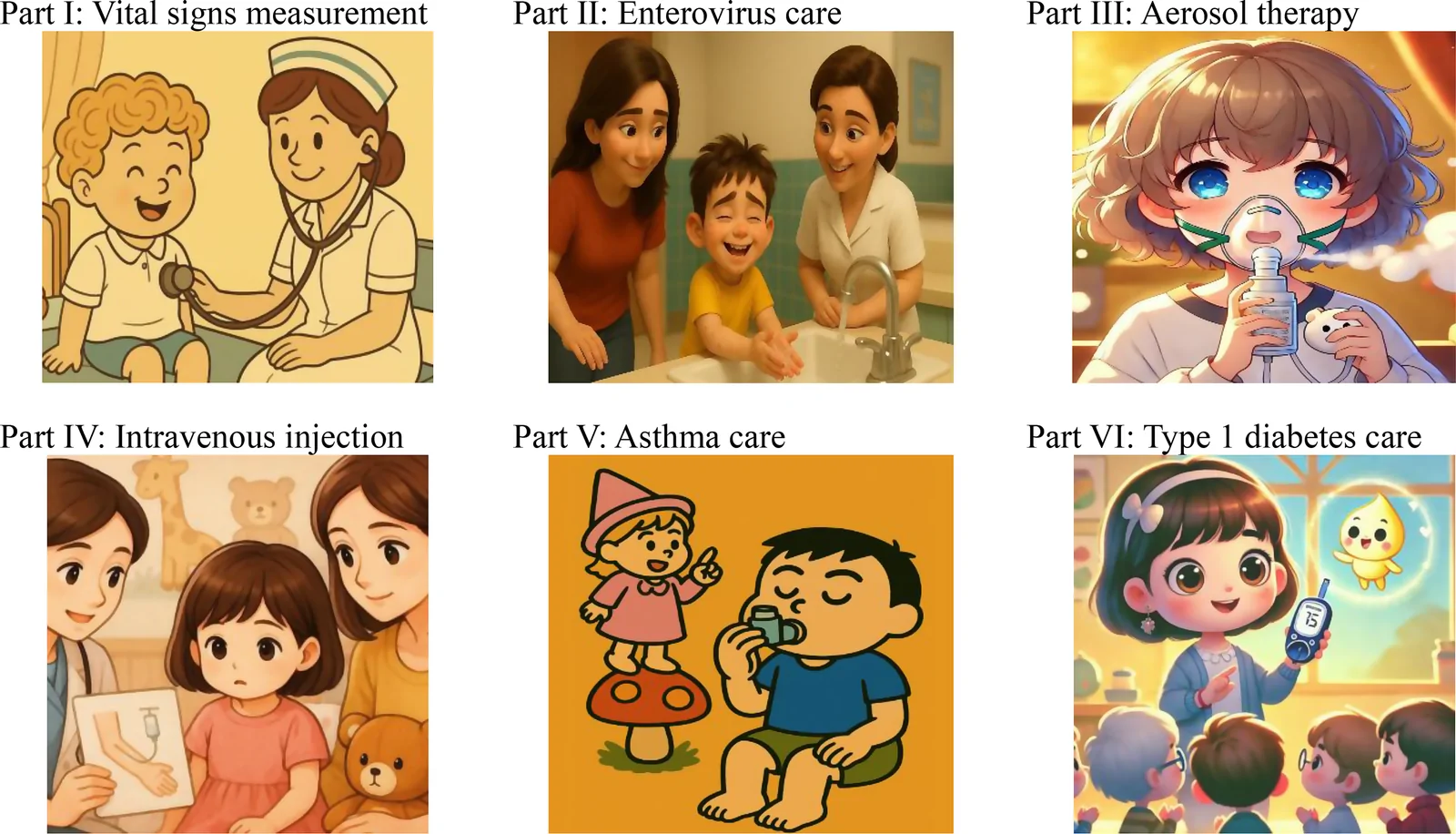
Six AI-generated pediatric medical e-picture books focusing on common nursing treatments

The intervention was conducted over seven instructional sessions, each lasting two hours. Under the guidance of the nursing instructor, students were organized into small groups of approximately 7–8 students. Each group developed AI-generated image-based e-picture books addressing six common pediatric examination and care topics: (1) vital signs measurement, (2) enterovirus care, (3) aerosol therapy, (4) intravenous injection, (5) asthma care, and (6) type 1 diabetes care (Fig. 1). Topic selection was aligned with pediatric nursing course objectives, common clinical practices in pediatric settings, and relevant literature. The content design emphasized procedural clarity, visual sequencing, and child-centered language to align with the developmental characteristics of pediatric patients. To ensure consistency and reproducibility, standardized prompting strategies were provided. The instructor introduced key principles, including role specification, audience targeting, task clarity, and iterative refinement. Students used structured prompts (e.g., explaining medical procedures to children in simple and reassuring language), with minor adaptations allowed to meet group needs. All prompts were developed under instructor guidance to ensure developmental appropriateness, clinical accuracy, and safety. All seven sessions were led by the same nursing instructor following a standardized session plan and slide deck, so that instructional content and pacing did not vary across groups. All groups began from an identical structured prompt template; the permitted adaptations were limited to substituting topic-specific medical content (e.g., procedure name, developmental stage of the target age group) and did not alter the underlying prompt structure or instructional goals. The same generative AI platform (Gemini) was used consistently by all groups across all seven sessions, and no alternative generative AI tools were substituted during the intervention period.

AI-generated outputs underwent a multi-stage review process. First, peer review was conducted within groups to assess clarity and relevance. Second, health-related content was verified against textbooks and evidence-based resources. Instructor feedback was provided iteratively, with particular attention to identifying inaccuracies or unsafe information, which students were required to revise. This process ensured clinical accuracy and patient safety. An evaluation rubric was developed to guide content quality, focusing on accuracy, clarity, and pediatric appropriateness. Expert validation by five specialists yielded a content validity index (CVI) of 1.00, indicating excellent content validity and alignment with professional nursing standards. Most creative and drafting work was completed collaboratively within groups; each student also completed a brief individual reflective task after each session to document personal engagement with the AI-assisted process, although the e-picture book itself remained a shared group product. Group e-picture books were formally scored against the evaluation rubric by the instructor at the end of the intervention, and rubric-based feedback, rather than a summative course grade, was used to guide the final round of revisions. AI-generated text and images were not used unedited: students manually reviewed and revised AI-generated outputs, in consultation with the instructor, to correct factual inaccuracies, adjust reading level, and ensure a supportive tone appropriate for pediatric patients, before outputs were finalized.

#### Technology acceptance

Technology acceptance was measured using a scale adapted from Lai and Ho (2025) [21], based on the Technology Acceptance Model proposed by Fred Davis (1989) [13]. The original instrument demonstrated satisfactory content validity (CVI = 0.85–0.88) and internal consistency (Cronbach’s α = 0.78–0.87) across five constructs: perceived usefulness (PU), perceived ease of use (PEOU), attitude toward use (ATU), behavioral intention (BI), and actual use (AU). The instrument was revised to fit the generative AI–assisted pediatric e-picture book learning context. Psychometric properties were re-evaluated in the current sample, showing good internal consistency (Cronbach’s α = 0.82–0.88 across subscales). The AU construct was defined as self-reported engagement in AI-assisted creation activities. Each of the five subscales comprises five items, with individual subscale scores ranging from 5 to 20 points; higher scores indicate greater acceptance of the technology. Because Actual Use was assessed via self-report rather than through objective behavioral or system-log data, it should be interpreted as a proxy for perceived engagement rather than as a direct behavioral measure. To reflect this measurement approach, the AU construct is referred to throughout this manuscript as self-reported (perceived) actual use rather than as a direct behavioral measure of system use.

The final scale included 25 items across the five constructs. All items were rated on a four-point Likert scale (1 = strongly disagree to 4 = strongly agree), with total scores ranging from 25 to 100; higher scores indicated greater acceptance. Content validity was confirmed by five experts (three nursing professors and two pediatric nurses), with CVI ≥ 0.80 [24]. Internal consistency reliability was assessed using Cronbach’s alpha, with values ≥ 0.80 indicating good reliability [25]. Item adaptation involved rewording the original chatbot-focused items [21] to refer specifically to the generative AI–assisted e-picture book creation task (e.g., substituting task-specific references to AI-generated pediatric health education content for the original HPV-education chatbot wording), while preserving the five-construct structure and the five-item-per-construct format. Because the source instrument was developed and validated in Mandarin Chinese with a comparable Taiwanese nursing student sample by two of the present co-authors [21], the adapted scale was administered in the same language without a separate translation step, and forward-backward translation procedures were therefore not applicable. A separate pilot test in an independent sample was not conducted for this adapted version; instead, item-level content validity was established through the five-expert panel review described above, with item wording revised iteratively until CVI ≥ 0.80 was achieved for every item. The full wording of the adapted 25-item scale is available from the corresponding author upon reasonable request.

The Actual Use (AU) subscale in the present study was operationalized as participants’ self-reported engagement with generative AI during the structured e-picture book creation activity, consistent with established TAM research employing post-task self-report measures as proxies for behavioral engagement [13, 21]. This approach is appropriate in educational settings where direct behavioral observation is not feasible; future studies are encouraged to supplement self-report measures with objective usage logs or instructor-assessed performance data. Notably, the correlation between BI and AU observed in this study (*r* = 0.97) was unexpectedly high, suggesting that the two constructs may not be empirically distinguishable when AU is operationalized through self-report.

### Ethics and data collection

This study was approved by the Research Ethics Committee (IRB No. 202400105A3). With the support of faculty members at the study site, the investigator introduced the study purpose, procedures, and participants’ rights to nursing students in classroom settings. Students were informed that participation was voluntary, that declining or withdrawing would not affect their academic standing, and that they could withdraw at any time without penalty. Written informed consent was obtained from students who agreed to participate prior to data collection, while those who declined were permitted to leave the classroom.

All participants were subsequently enrolled in a study-specific online group through which standardized instruction on the use of generative AI tools was provided, along with a rubric to guide group-based creation of pediatric medical care e-picture books. At the end of the semester, participants submitted their completed e-picture books and were invited to complete an anonymous online questionnaire. Informed consent information was presented on the first page of the questionnaire, and consent was implied by survey completion. The questionnaire required approximately 10–15 min to complete. Research data were collected anonymously, coded to protect participant identity, and stored in password-protected files accessible only to the research team. Confidentiality and data security were strictly maintained throughout the study to safeguard participants’ privacy.

### Data analysis

This study employed SPSS 26.0 (IBM Corp., Armonk, NY, USA) for all analyses. Continuous variables were summarized using means and standard deviations; categorical variables were reported as frequencies and proportions. Independent samples t-tests and one-way ANOVA examined group differences in TAM constructs. Pearson correlation analysis explored relationships among TAM variables, and a series of hierarchical linear regression analyses were conducted to estimate path coefficients. Prior to regression, statistical assumptions (including normality, linearity, homoscedasticity, and independence) were verified and confirmed. Multicollinearity was assessed using variance inflation factors (VIFs); all values remained below the conventional threshold (VIF < 10), confirming no problematic collinearity [26]. Demographic and technology-related variables (including academic year, clinical experience, daily digital device use, and generative AI use frequency) were included as control variables based on TAM theory and prior evidence indicating that these factors may shape technology perceptions and acceptance [13, 19, 27, 28]. Given the sample size (*N* = 80), which is insufficient to support stable structural equation modeling (SEM) estimation of the five-construct measurement and structural model, hierarchical linear regression was adopted as an exploratory, association-based analytic strategy rather than a formal causal or latent-variable modeling approach. Consequently, discriminant validity indices that require a measurement model (e.g., heterotrait-monotrait ratio, Fornell-Larcker criterion, average variance extracted) and latent-variable common method bias diagnostics (e.g., confirmatory factor analysis-based Harman’s single-factor test) could not be computed in the present analysis.

## Results

### Influence of external variables on TAM constructs among nursing students

A total of 80 nursing students were included in this study. The mean age of the participants was 24.86 years (SD = 2.80). Regarding gender distribution, the majority of participants were female (78.7%, *n* = 63), while males accounted for 21.3% (*n* = 17). In terms of academic year, most participants were third-year students (83.7%, *n* = 67), followed by fourth-year students (11.3%, *n* = 9), whereas first-year students comprised a smaller proportion of the sample (5.0%, *n* = 4). No second-year students were included. This distribution indicates that most participants were at a stage of advanced professional coursework and increasing clinical training.

With respect to clinical nursing experience, 39.0% of participants (*n* = 57) reported having clinical nursing experience, whereas 61.0% (*n* = 23) reported no prior clinical experience, suggesting that a substantial proportion of the sample had begun exposure to clinical practice. Regarding daily digital device use, the largest proportion of participants reported using digital devices for 3–5 h per day (38.8%, *n* = 31), followed by 1–3 h (23.8%, *n* = 19) and 5–8 h (17.5%, *n* = 14). Additionally, 12.5% (*n* = 10) reported using digital devices for more than 10 h per day, indicating a high level of overall digital engagement among participants.

In terms of generative AI use frequency, most participants reported using generative AI several times per week (70.0%, *n* = 56), suggesting that generative AI had become a routine tool in their daily learning or personal activities. This was followed by once-daily use (12.5%, *n* = 10). In contrast, only 10.0% (*n* = 8) reported never using generative AI, while 7.5% (*n* = 6) reported using generative AI multiple times per day. Detailed participant characteristics are presented in Table 1.

**Table 1:** Descriptive statistics of participants’ demographic characteristics and TAM constructs

Variable	*N* (%)	Mean ± SD
**Age (years)**		24.86 ± 2.80
**Sex**		
Male	17 (21.3)	
Female	63 (78.7)	
**Year level**		
First year	4 (5.0)	
Second year	0 (0.0)	
Third year	67 (83.7)	
Fourth year	9 (11.3)	
**Clinical Nursing Experience**		
No Experience	23 (61.0)	
Have Experience	57 (39.0)	
**Daily Time Spent Using Digital Devices** *(including computers*,* smartphones*,* or pad)*		
Less than 1 h	2 (2.5)	
1–3 h	19 (23.8)	
3–5 h	31 (38.8)	
5–8 h	14 (17.5)	
8–10 h	0 (5.0)	
More than 10 h	10 (12.5)	
**Frequency of Using Generative AI** *(e.g.*,* ChatGPT*,* AI-Generated Images*,* AI-Generated Short Videos*,* etc.)*		
Never use	8 (10.0)	
Use several times per week	56 (70.0)	
Use once per day	10 (12.5)	
Use multiple times per day	6 (7.5)	

(*N* = 80)

### Correlations among the five dimensions of using generative AI to create e-picture books

Table 2 shows that the descriptive statistics and Pearson correlation coefficients among the five TAM dimensions PU, PEOU, ATU, BI, and AU in the context of nursing students’ use of generative AI to create pediatric medical care e-picture books. The mean scores for the five dimensions ranged from 17.05 to 17.55, indicating that nursing students generally held positive perceptions and a moderate to high level of acceptance toward using generative AI for the design and development of pediatric medical care e-picture books. Correlation analysis revealed strong and statistically significant positive associations among all five dimensions (*r* = 0.86–0.98, *p* < 0.01). Specifically, PU was significantly correlated with PEOU (*r* = 0.88), ATU (*r* = 0.86), BI (*r* = 0.86), and AU (*r* = 0.90). These uniformly high correlations (all *r* > 0.85) are noted here as a methodological consideration regarding discriminant validity and potential common method variance.

**Table 2:** Correlations among five dimensions of generative AI acceptance for developing health education tools

Variables	Mean ± SD	Range	1	2	3	4	5
			*r*	*r*	*r*	*r*	*r*
1	17.55 ± 2.24	15–20	1				
2	17.05 ± 2.29	13–20	0.88**	1			
3	17.30 ± 2.50	14–20	0.86**	0.90**	1		
4	17.34 ± 2.38	14–20	0.86**	0.90**	0.98**	1	
5	17.40 ± 2.50	14–20	0.90**	0.94**	0.97**	0.97**	1

Row and column headings: 1 = Perceived usefulness, 2 = Perceived ease of use, 3 = Attitude toward use, 4 = Behavioral intention to use, 5 = Actual use.Pearson correlation coefficient. *p < 0.05, **p < 0.01, ***p < 0.001.*N* = 80

In addition, PEOU showed strong positive correlations with ATU (*r* = 0.90), BI (*r* = 0.90), and AU (*r* = 0.94), highlighting the critical role of usability and low learning barriers in fostering positive attitudes and sustained intention to use generative AI during the e-picture book creation process. Notably, the correlation between ATU and BI was extremely high (*r* = 0.98, *p* < 0.01), and BI was also strongly correlated with AU (*r* = 0.97, *p* < 0.01).

### Acceptance of using generative AI to create pediatric medical care e-picture books

Table 3 shows that the results of the linear regression analyses conducted to examine factors influencing nursing students’ acceptance of using generative AI to create clinical health education tools, with pediatric medical care e-picture books serving as the specific application context. Regarding PU, the overall regression model was statistically significant and demonstrated high explanatory power (R² = 0.809). PEOU showed the strongest positive association with PU (β = 0.878, *p* < 0.001); nursing students who perceived generative AI as easy to operate and associated with a low learning burden were also more likely to report it as beneficial for designing pediatric medical care e-picture books and supporting clinical health education communication.

**Table 3:** Regression analysis of factors influencing acceptance of generative AI for developing health education tools

DV	*R* ^2^	IV	B	SE	β	95% CI	t	*p*
Perceived usefulness	0.809 (*F* = 37.517, *p* < 0.001)	Constant	3.753	1.048		1.664 to 5.843	3.582	0.001**
		First year^a^	-2.074	0.701	-0.203	-3.473 to -0.675	-2.957	0.004**
		Third year^a^	-1.044	0.439	-0.173	-1.918 to -0.169	-2.380	0.020*
		Less than 1 hour^b^	-0.923	0.848	-0.065	-2.613 to 0.767	-1.089	0.280
		3–5 hours^b^	0.354	0.315	0.078	-0.274 to 0.983	1.124	0.265
		5–8 hours^b^	-0.119	0.387	-0.020	-0.891 to 0.652	-0.308	0.759
		8–10 hours^b^	-0.450	0.591	-0.044	-1.629 to 0.729	-0.761	0.449
		More than 10 hours^b^	0.532	0.439	0.079	-0.343 to 1.408	1.212	0.229
		Perceived ease of use	0.858	0.056	0.878	0.746 to 0.971	15.252	< 0.001***
Perceived ease of use	0.186 (*F* = 2.358, *p* < 0.05)	Constant	16.994	0.943		15.114 to 18.875	18.015	< 0.001***
		First year^a^	-1.452	1.459	-0.139	-4.360 to 1.456	-0.995	0.323
		Third year^a^	0.225	0.918	0.037	-1.604 to 2055	0.246	0.807
		Less than 1 hour^b^	3.006	1.797	0.206	-0.576 to 6.587	1.673	0.099
		3–5 hours^b^	0.208	0.660	0.039	-1.107 to 1.523	0.316	0.753
		5–8 hours^b^	-0.934	0.703	-0.156	-2.335 to 0.468	-1.328	0.188
		8–10 hours^b^	2.280	1.153	0.219	-0.018 to 4.579	1.978	0.052
		More than 10 hours^b^	-1.084	0.804	-0.158	-2.686 to 0.518	-1.349	0.182
Attitude toward use	0.828 (*F* = 477.22, *p* < 0.001)	Constant	-0.432	0.944		-2.311 to 1.447	-0.458	0.649
		Perceived usefulness	0.364	0.111	0.326	0.144 to 0.585	3.289	0.002**
		Perceived ease of use	0.665	0.108	0.609	0.449 to 0.881	6.140	< 0.001***
Behavioral intention to use	0.952 (*F* = 761.50, *p* < 0.001)	Constant	0.923	0.470		-0.012 to 1.859	1.965	0.053
		Perceived usefulness	0.090	0.052	0.084	-0.015 to 0.194	1.709	0.091
		Attitude toward using	0.858	0.047	0.902	0.764 to 0.951	18.275	< 0.001***
Actual use	0.943 (*F* = 1279.71, *p* < 0.001)	Constant	-0.338	0.500		-1.335 to 0.658	0.676	0.501
		Behavioral intention to use	1.023	0.029	0.971	0.966 to 1.080	35.773	< 0.001***

Reference group: ^a^ Fourth year; ^b^ 1–3 h. (*N* = 80)

Among background variables, first-year students (β = −0.203, *p* = 0.004) and third-year students (β = −0.173, *p* = 0.020) reported significantly lower PU compared with fourth-year students, whereas daily digital device use time did not significantly predict PU (*p* > 0.05). In contrast, for PEOU, the overall model reached statistical significance but demonstrated relatively lower explanatory power (R² = 0.186). None of the background variables, including academic year and daily digital device use time, were significant predictors (all *p* > 0.05), suggesting that nursing students’ perceptions of the usability of generative AI were relatively independent of individual background characteristics. For ATU, the regression model showed strong explanatory power (R² = 0.828). Both PU (β = 0.326, *p* = 0.002) and PEOU (β = 0.609, *p* < 0.001) were significantly associated with ATU; nursing students who perceived generative AI as both useful and easy to use were also more likely to report positive attitudes toward its use. In terms of BI, the regression model demonstrated exceptionally high explanatory power (R² = 0.952). ATU showed the strongest and only significant association (β = 0.902, *p* < 0.001), whereas PU did not reach statistical significance (*p* = 0.091). This finding suggests that, in the context of this study, nursing students’ intention to continue using generative AI was most strongly associated with their overall attitudes toward the technology. Given the pronounced imbalance in academic-year subgroup sizes (first-year, *n* = 4; fourth-year, *n* = 9; third-year, *n* = 67; no second-year students), the year-level comparisons reported above should be interpreted with caution and not generalized across cohorts.

Finally, in the analysis of AU, the regression model also exhibited high explanatory power (R² = 0.943). BI showed a highly significant and very strong association with AU (β = 0.971, *p* < 0.001). Given that AU was operationalized via self-report rather than objective behavioral data, this exceptionally strong association likely reflects, at least in part, conceptual and measurement overlap between BI and AU rather than solely a behavioral translation of intention into engagement.

## Discussion

Several background characteristics of the nursing student sample provide useful context for interpreting the study findings, although given the pronounced imbalance in academic-year subgroup sizes (Table 1), any patterns associated with year of study are reported here strictly as exploratory, sample-specific associations rather than as evidence of a developmental or educational effect. With that caveat, students who had accumulated more coursework and clinical exposure may, in principle, be differently positioned to evaluate emerging educational technologies such as generative AI [29–31]; whether this pattern reflects a genuine developmental trend cannot be established from the present, unevenly distributed sample and would require confirmation in a cohort with balanced representation across all four academic years.

Clinical exposure may further shape how students perceive the applicability of generative AI. Prior research suggests that early clinical experiences enhance awareness of real-world challenges, such as time constraints in patient education and communication barriers with children and caregivers [32]. In this context, students with clinical experience may be more likely to recognize the practical value of AI-assisted tools, whereas those without such experience may engage with these technologies at a more exploratory level. Although nursing students are generally familiar with digital technologies, the Technology Acceptance Model indicates that general digital experience alone does not ensure acceptance. Instead, perceived usefulness and ease of use within a specific learning context are more critical determinants [30]. Variations in prior experience with generative AI may also contribute to differences in engagement, reflecting individual readiness and perceived task relevance. Overall, these findings suggest that acceptance of generative AI is influenced by learners’ stage of professional development and contextual readiness rather than by uniform exposure to technology. This highlights the importance of aligning AI-supported educational interventions with students’ learning needs and clinical preparedness [28]. Interrelationships Among TAM Constructs in Generative AI–Assisted Health Education.

The results of this study indicate that all five TAM constructs PU, PEOU, ATU, BI, and AU were highly and significantly positively correlated. This finding suggests that nursing students’ evaluations of generative AI are highly integrated: students who perceived generative AI as easy to use were also more likely to regard it as useful, hold positive attitudes, and report stronger intentions to use the technology, which were in turn associated with self-reported actual usage. However, the uniformly high correlations among constructs (*r* = 0.86–0.98) also raise the possibility that common method variance inflated the observed associations, a well-documented risk in single-source, single-occasion self-report designs [33], in addition to raising concerns about limited discriminant validity among the TAM constructs. These findings are consistent with recent studies applying TAM to generative AI or chatbot technologies in higher education and nursing education contexts [34–36].

Furthermore, the mean scores for all TAM constructs were relatively high, indicating that participants generally held positive attitudes toward generative AI use in pediatric healthcare education. Overall, these findings support the theoretical assumption that technologies are more likely to be holistically accepted when they simultaneously satisfy task requirements and provide a positive user experience [13].

The finding that PEOU showed the strongest association with PU, and that ATU was most strongly associated with BI, is consistent with the core TAM behavioral pathway. Of particular interest is the prominent role of affective attitudes (ATU) in this educational context. Generation Z nursing students are generally comfortable with digital tools; however, acceptance of a specific AI application within a task-based context appears to be more strongly shaped by emotional engagement and perceived fit than by prior digital familiarity alone. When students found generative AI intuitive and effective for creating pediatric health education materials, the resulting experience of competence and productivity likely reinforced both their positive attitudes and their intentions to continue using the technology [13, 36]. The structured, task-embedded nature of the intervention may have further amplified the role of ATU as a bridge between cognitive appraisals and behavioral intentions, suggesting that in voluntary, task-based educational contexts, affective responses serve as a primary motivational mechanism for continued engagement. Usability appears to play a central role in shaping students’ perceptions of value, consistent with prior research in nursing and health professions education highlighting the importance of minimizing cognitive and technical barriers to enhance perceived usefulness and affective engagement [27, 37].

The results further indicate that perceptions of usability may be relatively stable across student subgroups, implying that well-designed generative AI tools can remain accessible regardless of learners’ prior digital experience or level of training. This aligns with previous studies showing that general digital familiarity alone does not guarantee acceptance of specific educational technologies [38]. Instead, acceptance is more strongly influenced by how well the technology aligns with task demands and learning contexts [39]. In addition, affective responses appear to play a critical role in shaping students’ intentions to use generative AI. Consistent with TAM-based research, attitudes toward use function as a key mechanism linking perceptions of usefulness and usability to continued engagement [27]. This highlights the importance of designing learning experiences that not only emphasize functionality but also foster positive user experiences.

Situating these findings within the broader literature on generative AI adoption in higher education, the prominent role of perceived ease of use and attitude toward use observed here parallels patterns reported for ChatGPT adoption among doctoral students completing academic writing tasks [40] and among nursing students engaging with generative AI more broadly [41], where usability and affective response likewise emerged as central drivers of continued use. This convergence across markedly different generative AI platforms and tasks, ranging from general-purpose chatbots such as ChatGPT, Gemini, and Copilot [8] to the task-specific, image-generation-based application examined here, suggests that TAM’s core usability-attitude-intention pathway may generalize across generative AI tools even as their specific capabilities and interfaces differ. At the same time, prior nursing-education TAM studies of simulation-based and chatbot-based technologies [18, 20, 21] have typically reported comparatively lower inter-construct correlations than those observed in the present study; this discrepancy may reflect differences in task structure or sample homogeneity, or may instead reflect the single-occasion, self-report design discussed above, rather than a substantively different acceptance process.

Overall, the findings of this study indicate that nursing students demonstrated a positive level of acceptance toward the use of generative AI in developing pediatric medical care e-picture books, supporting the applicability of the Technology Acceptance Model in this educational context. The relationships among PEOU, PU, ATU, BI, and AU were consistent with established TAM pathways, suggesting that usability and perceived value play central roles in shaping students’ engagement with AI-assisted learning tools. Importantly, the results should be interpreted within the scope of an educational application. While the use of generative AI–assisted e-picture books may be relevant to the development of competencies related to pediatric health education and communication, this study did not directly evaluate clinical performance, nurse–child interaction, or patient-related outcomes. Therefore, any potential implications for clinical practice should be considered cautiously and warrant further empirical investigation in real-world healthcare settings.

### Theoretical implications

This study applies the Technology Acceptance Model to a new context—generative AI–assisted learning in pediatric nursing education. The results show that students are more likely to accept this technology when they find it easy to use and useful. In addition, students’ attitudes toward using the technology play an important role in shaping their intention to continue using it, especially in learning situations where use is voluntary. Furthermore, this study places the Technology Acceptance Model within a specific learning task, namely the creation of pediatric health education materials. The findings suggest that technology acceptance is not only influenced by students’ general familiarity with digital tools, but also by how well the technology fits the learning task and the students’ level of professional preparation. Overall, these results highlight that both the learning context and students’ experience are important factors in understanding how generative AI is accepted in nursing education.

### Practical implications

The findings of this study highlight the potential value of integrating generative AI into pediatric nursing education, particularly when students perceive the technology as both easy to use and useful. Based on these results, nursing educators are encouraged to incorporate structured AI-assisted learning activities into curricula, such as guided e-picture book creation tasks, which may offer a promising avenue for building pediatric health communication skills; this potential was not directly tested in the present acceptance-focused study and warrants dedicated outcome evaluation. Providing clear instructions, example prompts, and iterative feedback may further enhance students’ engagement with such activities.

In addition, the strong role of perceived ease of use suggests that selecting user-friendly AI tools and offering initial training or scaffolding are critical for successful implementation. Educational programs should therefore prioritize intuitive platforms and provide technical support to reduce cognitive burden and facilitate adoption. These strategies align with current trends in digital health education, where usability and task alignment are key factors influencing effective learning. From a practical perspective, AI-assisted e-picture books may serve as supplementary educational materials in pediatric settings, particularly for explaining procedures or health concepts to children in a more engaging and developmentally appropriate manner. However, as this study focused on students’ acceptance rather than clinical outcomes, such applications should be implemented with caution. Human verification, alignment with evidence-based guidelines, and professional oversight remain essential to ensure accuracy and patient safety [42].

Overall, these findings provide preliminary guidance for educators by emphasizing the importance of usability, instructional design, and contextual alignment when integrating generative AI into pediatric nursing education.

### Limitations and future research recommendations

This study applied the Technology Acceptance Model to examine nursing students’ acceptance of generative AI within a clearly defined educational context. Participants engaged in hands-on AI-assisted e-picture book creation prior to data collection, ensuring that responses reflected actual user experience.

Several limitations should be noted. The sample was drawn from a single institution and included students who had completed Human Development and Pediatric Nursing courses, supporting alignment with the study objectives. Students lacking basic computer skills were excluded to ensure valid interaction with generative AI tools. All eligible students were invited to participate to reduce selection bias. The predominance of third-year students reflects the curriculum structure, as they had completed relevant coursework and clinical exposure. However, the single-institution design and uneven distribution across academic years may limit representativeness and generalizability. In addition, clinical outcomes were not assessed; thus, findings reflect acceptance of an educational application rather than clinical effectiveness. Future studies with multi-institutional samples and objective outcome measures are recommended. Additionally, the single-source, cross-sectional design introduces the potential for common method bias (CMB). Although procedural remedies (including anonymous data collection, post-task questionnaire administration, and the use of a validated instrument) were implemented, a formal CMB test (e.g., Harman’s single-factor analysis or Kock’s full collinearity VIF diagnostics via PLS-SEM) was not conducted. Consistent with established guidance on common method bias [33], this omission should be considered when interpreting the uniformly high correlations among TAM constructs reported in the Results, as some portion of these associations may reflect shared method variance rather than substantive covariation alone. Future studies are encouraged to employ structural equation modeling frameworks and report comprehensive CMB diagnostics to strengthen methodological rigor.

Several additional methodological limitations warrant explicit acknowledgment. Because the sample size (*N* = 80) was insufficient to support stable full structural equation modeling, this study relied on correlation and hierarchical regression analyses rather than SEM, which is the more conventional TAM analytic approach; consequently, structural path coefficients should be interpreted as associations among manifest scale scores rather than as latent structural relationships, and future research with larger samples should employ SEM (e.g., via AMOS, LISREL, SmartPLS, or Mplus) to validate these pathways. The correlation between Behavioral Intention and Actual Use was exceptionally high (*r* = 0.97), and the Actual Use measure relied on post-intervention self-report rather than observed behavior; this indicates that the two constructs may not be empirically distinguishable in the present data, and future studies should incorporate objective indicators of system use (e.g., platform usage logs, prompt histories, instructor-rated engagement) to more clearly differentiate intention from actual behavior. All five TAM constructs were correlated above *r* = 0.85, which exceeds conventional thresholds for discriminant validity; because the analytic approach used manifest scale-level correlations rather than a confirmatory factor-analytic measurement model, indices such as the heterotrait-monotrait ratio, Fornell-Larcker criterion, average variance extracted, and CFA-based Harman’s single-factor test could not be computed, and this should be considered an important limitation when interpreting the strength of associations among constructs. In addition, because a stable exploratory factor analysis could not be conducted at the achieved sample size (see Data Analysis) [43], the assumption that the 25 items load onto five empirically distinct factors, rather than a smaller number of overlapping factors, could not be directly verified in this study and should be treated as a working assumption inherited from the source instrument [21] rather than as an independently confirmed property of the adapted scale. Finally, the adapted TAM scale was not piloted in an independent sample prior to full administration, and its full item wording is not reproduced within the manuscript; future replications should include a formal pilot phase and publish the complete instrument as a supplementary appendix. The sample was markedly imbalanced across academic years (83.7% third-year, 11.3% fourth-year, 5.0% first-year, and no second-year students); year-level comparisons reported in the Results and Discussion should therefore be interpreted with caution and not treated as robust cross-cohort comparisons.

To address accuracy and safety, AI-generated content was verified against evidence-based resources, supported by instructor oversight and iterative revision. Future applications should further incorporate ethical guidelines and AI literacy training. Future studies employing structural equation modeling frameworks are also encouraged to conduct comprehensive common method bias diagnostics (e.g., Kock’s full collinearity test) and report full-model fit indices to further validate the TAM pathways identified in this study.

## Conclusion

This study demonstrated that nursing students exhibited a high level of acceptance of using generative AI to create pediatric health education e-picture books, providing preliminary, association-based support for the applicability of the Technology Acceptance Model in this educational context that should be confirmed through structural equation modeling in future, adequately powered samples. The findings highlight the role of perceived ease of use and perceived usefulness in shaping students’ attitudes, behavioral intentions, and actual use of generative AI. The findings also suggest that integrating generative AI into nursing education may support innovative learning approaches and contribute to the development of digital competencies among nursing students. This study evaluated technology acceptance rather than educational or clinical effectiveness. While generative AI–assisted e-picture book creation represents a promising approach in nursing education, its impact on clinical practice, child comprehension, and patient outcomes was not examined in this study and should be explored in future research.

## Data Availability

Data available on request due to restrictions regarding privacy, legal and ethical concerns. The data presented in this study are available on request from the corresponding author.
